# The Impact of Perioperative Anaesthesia Management on Cancer Recurrence

**DOI:** 10.5152/TJAR.2022.22176

**Published:** 2022-10-01

**Authors:** Oscar Díaz-Cambronero, Pilar Argente Navarro

**Affiliations:** 1 Department of Anaesthesiology, Hospital Universitari i Politécnic la Fe, Valencia, Spain; 2 Perioperative Medicine Research Group, Instituto de Investigación Sanitaria la Fe, Valencia, Spain; 3 EuroPeriscope, Oncoanaesthesiology Research group, European Society Anaesthesiology and Intensive Care (ESA-IC)

Cancer is the second leading cause of mortality and disability all over the world.^1^ Cancer therapy including neoadjuvant chemotherapy and/or radiotherapy is complex, but surgery remains the main curative treatment for solid tumours and almost 80% of cancer types will need surgery. Despite curative intention, surgery is unavoidable and is associated with tumour cell liberation into circulation, and inflammatory response. There is a growing body of evidence supporting that the surgical inflammatory response related to surgery could be linked to different mechanisms leading to cancer recurrence and poor long-term outcomes.^2,3^ This paradoxical effect has been postulated and mediated by immunosuppression, proangiogenic state, and directly by inflammatory response.^4^ Surgical cancer manipulation leads to liberation of circulating tumour cells with potential for metastasis, and a high number of circulating tumour cells are related to poor prognosis.^5^ The progression from circulating tumour cells to clinical recurrence mainly depends on evasion of host´s immunocompetence defence and tumour angiogenesis. Thus, perioperative management of surgical oncological patients seems to be cornerstone to maintain immunocompetence and potentially modulate recurrence or metastasis.^6,7^

Anaesthetic management and perioperative care have radically evolved in the last decades due to “fast track” concept and enhanced recovery pathways, with the goal of improving patient recovery from surgery by modulating surgical aggression and perioperative management. In the last few years, the focus has moved to long-term outcomes, patient-reported outcomes, and experience. This personalised medicine with a holistic, integral, multidisciplinary perioperative management and patient engagement is the concept of perioperative medicine.^8-10^ Perioperative medicine in oncological patients or oncoanaesthesia has become an excellent model to explore the impact of perioperative care interventions on long-term outcomes.^11-15^ The approach has been initially to explore which could be the best anaesthetic technique such as propofol versus halogenated agents, and opiates versus regional anaesthetic techniques. Nonetheless, in the last few years, the introduction of pharmacological interventions like lidocaine intravenous infusion or immunotherapeutic agents in the perioperative period has also become an important focus for research on other non-pharmacological interventions.

There are preclinical data supporting that some standard anaesthetic interventions like halogenated drugs or systemic opiates could be associated with worse oncological outcomes due to proangiogenic, immunosuppressive, or pro-inflammatory effects.^16-18^ On the other hand, there are some anaesthetic interventions like propofol, lidocaine, dexmedetomidine, or COX-2 antagonists that might be associated with a better oncological outcome.^19-21^ These results are sometimes conflicting and should be interpreted with caution due to difficulties in extrapolating data from an in vitro or animal model to the complex environment in the perioperative setting; however, they are useful for the generation of hypotheses for clinical research.

At the clinical level, the evidence is even more conflicting and mainly related to pharmacological interventions in retrospective studies or database analysis. The use of halogenated agents versus propofol has been extensively studied,^22-24^ and despite a trend for better long-term outcomes with propofol in the different meta-analyses, no high-quality evidence is available. Opioids have also been linked to poor outcomes,^25-27^ but despite an increase in some opioid receptor expression in several types of cancer tissue, association with worse outcomes is also conflicting depending on cancer type.^28-35^ The use of regional anaesthesia has also been suggested as a potential intervention but is even more elusive to evaluate because it is very difficult to isolate the impact on pain relief from the effect of local anaesthesia by itself. The non-steroidal anti-inflammatory drugs (NSAIDs) have also been tested, but a single administration of ketorolac tromethamine before surgery does not increase disease-free survival in high-risk breast cancer patients.^36,37^

Non-pharmacological interventions such as prehabilitation, immunonutrition, anxiety relief, temperature homeostasis, or blood transfusion saving have also been proposed, but no high-quality evidence is available on the long-term impact. Unfortunately, the most important randomised clinical trial in breast cancer testing the impact of the interventional paravertebral block and propofol versus volatile anaesthesia with sevoflurane and opioids did not reduce cancer recurrence after potentially curative surgery.^38^ Future studies such as registry-based randomised trials, high-quality observational registries or pragmatic randomised trials are required to definitely answer this question.

We must acknowledge the complex interplay of oncological surgery and understand that there is no magic bullet to improve long-term outcomes. Individual tumor targeted chemotherapy and radiotherapy, which is designed according to the genetic type of the tumor and the patients’ condition, achieve the best results and have been suggested to be used in the more sensitive perioperative period.^39^ In perioperative medicine for oncological patients, we are still far from this personalised medicine, and negative research findings looking for a size that fits all could not be surprising.

For now, there is no high quality evidence which may change the perioperative management of oncologic patients. There are several reasons that probably explain this lack of evidence, including testing single interventions in an overly simplistic model with the goal of achieving long-term outcomes; mainly retrospective analysis or unplanned analysis of clinical trials; optimal control of inflammatory response related to surgery; lack of standardised endpoints in oncoanaesthesia; inclusion of low-recurrence types of cancer; and mixed types or stages of cancer in many studies.^40^

There is enough rationale to support that any intervention in the perioperative period could have an impact on long-term outcomes in the oncological patient, but negative results also pointed out that the more aggressive the surgery and the cancer type, the more effective the interventions. This means that we are on the right path and the perioperative holistic intervention must consider all the factors that may potentially facilitate tumour progression, including perioperative analgesia and control of sympathetic response (regional anaesthesia, local anaesthetics, opioids, NSAIDs, hypnotic agents, temperature homeostasis, blood transfusion, nutrition status, exercise, or pain anxiety) to improve patient immunocompetence, maintain patient homeostasis, and modulate the surgical stress response to avoid cancer progression.^9,41-43^

Nonetheless, this is a worthy challenge, and perioperative oncological medicine will provide targeted interventions in the near future.
